# Prkaa1 Metabolically Regulates Monocyte/Macrophage Recruitment and Viability in Diet-Induced Murine Metabolic Disorders

**DOI:** 10.3389/fcell.2020.611354

**Published:** 2021-01-12

**Authors:** Qiuhua Yang, Qian Ma, Jiean Xu, Zhiping Liu, Jianqiu Zou, Jian Shen, Yaqi Zhou, Qingen Da, Xiaoxiao Mao, Sarah Lu, David J. Fulton, Neal L. Weintraub, Zsolt Bagi, Mei Hong, Yuqing Huo

**Affiliations:** ^1^Vascular Biology Center, Department of Cellular Biology and Anatomy, Medical College of Georgia, Augusta University, Augusta, GA, United States; ^2^State Key Laboratory of Chemical Oncogenomics, Key Laboratory of Chemical Genomics, School of Chemical Biology and Biotechnology, Peking University Shenzhen Graduate School, Shenzhen, China; ^3^Department of Cardiology, Second Affiliated Hospital of Zhejiang University School of Medicine, Hangzhou, China; ^4^Department of Cardiovascular Surgery, Peking University Shenzhen Hospital, Shenzhen, China; ^5^Trinity College of Arts & Sciences, Duke University, Durham, NC, United States; ^6^Department of Physiology, Medical College of Georgia, Augusta University, Augusta, GA, United States

**Keywords:** AMPKα1/PRKAA1, glycolysis, monocyte recruitment, macrophage viability, metabolic disorders

## Abstract

Myeloid cells, including monocytes/macrophages, primarily rely on glucose and lipid metabolism to provide the energy and metabolites needed for their functions and survival. AMP-activated protein kinase (AMPK, its gene is *PRKA* for human, *Prka* for rodent) is a key metabolic sensor that regulates many metabolic pathways. We studied recruitment and viability of *Prkaa1*-deficient myeloid cells in mice and the phenotype of these mice in the context of cardio-metabolic diseases. We found that the deficiency of Prkaa1 in myeloid cells downregulated genes for glucose and lipid metabolism, compromised glucose and lipid metabolism of macrophages, and suppressed their recruitment to adipose, liver and arterial vessel walls. The viability of macrophages in the above tissues/organs was also decreased. These cellular alterations resulted in decreases in body weight, insulin resistance, and lipid accumulation in liver of mice fed with a high fat diet, and reduced the size of atherosclerotic lesions of mice fed with a Western diet. Our results indicate that AMPKα1/PRKAA1-regulated metabolism supports monocyte recruitment and macrophage viability, contributing to the development of diet-induced metabolic disorders including diabetes and atherosclerosis.

## Introduction

Metabolic syndrome, including obesity and diabetes as well as their vascular complications such as atherosclerosis, are chronic inflammatory diseases (Ross, 1999; Libby, 2002; Weisberg et al., 2003; Xu et al., 2003; Lumeng and Saltiel, 2011). These diseases are initiated by recruitment of circulating leukocytes, including monocytes, T cells, neutrophils, and NK cells, to the metabolic organs/tissues and vascular walls (Ley et al., 2007; Shi and Pamer, 2011). The infiltrated leukocytes survive in the affected organs/tissues, modulate their activation status and subsequently conquer the progression of these metabolic disorders and their complications. The importance of leukocyte recruitment and activation in the development and progression of these diseases has been demonstrated in various studies in which disease development is suppressed by genetic or pharmacologic blockade of leukocyte recruitment molecules such as selectins, selectin ligands, integrins, ligands for integrins, chemokines and chemokine receptors (Ley et al., 2007; Wu and Ballantyne, 2020). Furthermore, reduction of survival and activation of these infiltrated leukocytes are also able to inhibit these metabolic disorders and their complications (Murray and Wynn, 2011).

The role of cellular metabolism in redirecting the fate of leukocytes and their activities has attracted interest in the field. A large number of studies focused on the metabolic status of leukocytes and its dynamic changes during homeostasis and inflammation and highlighted that the metabolic status of leukocytes can undergo reprogramming in response to environmental stimuli (Webb et al., 2017; Marelli-Berg and Jangani, 2018). Aerobic glycolysis appears to be the most important metabolic pathway in leukocytes to produce ATP rapidly and sufficiently to support leukocyte recruitment and migration. It has been reported that macrophage HIF1α deficiency significantly suppressed migration under hypoxia conditions, and the results suggest that glycolysis sustains macrophage migration into inflamed tissues (Semba et al., 2016). In addition to macrophage migration, it has been demonstrated that macrophages largely depend on glucose supply through glycolysis-induced ATP production in cell spreading and protrusion formation (Venter et al., 2014). However, less is known about the impact of metabolic alterations on leukocyte recruitment and survival in diet-induced metabolic syndrome.

AMPK/PRKA acts as a major cellular energy sensor and a master regulator of metabolic homeostasis, including regulating the signals involved in glucose, lipid and cholesterol metabolism (Hardie and Hawley, 2001; Hardie, 2004). In mammals, AMPK/PRKA exists as a conserved serine/threonine kinase comprised of a catalytic subunit (α) and two regulatory subunits (β and γ). AMPKα1/PRKAA1 is the predominant isoform found in vascular cells and monocytes/macrophages, while AMPKα2/PRKAA2 is the catalytic isoform expressed in the liver, muscle and hypothalamus (Kahn et al., 2005; Fisslthaler and Fleming, 2009; Hardie, 2011). A few groups have extensively studied the role of AMPKα1/PRKAA1 in regulating cardiovascular functions and the development of cardiovascular diseases (Cai et al., 2016; Ding et al., 2017; Wu and Zou, 2020). In most of these studies, the kinase function of AMPKa1/PRKAA1 has been emphasized. AMPKa1/PRKAA1 is a well-characterized molecule for modulating cellular metabolism, including metabolism of glucose and fatty acid oxidation, in metabolic tissue/organs (Garcia and Shaw, 2017; Boudaba et al., 2018; Wu et al., 2018). For cardiovascular cells, we recently examined the role of AMPKa1/PRKAA1/Prkaa1 in glucose metabolism of cultured human endothelial cells and mouse endothelial cells *in vivo* (Yang et al., 2018); the role of AMPKa1/PRKAA1/Prkaa1 in macrophage metabolism, such as glucose metabolism, has not been studied. Here, we examined glycolysis in *Prkaa1*-deficient myeloid cells and the effects of this changed metabolism to myeloid cell recruitment and survival, as well as to the development of chronic inflammatory diseases, including diet-induced diabetes and atherosclerosis.

## Materials and Methods

### Mouse Generation and Breeding

*Prkaa1*-floxed (*Prkaa1*^Lox/Lox^, *Prkaa1*^WT^) mice were kindly provided by Dr. Benoit Viollet (Institut Cochin, Paris, France). These mice were first crossed with myeloid-specific Cre recombinase-expressing mice (*Lysm*^Cre/Cre^ mice, Cat. No. 004718, the Jackson Laboratory, Bar Harbor, ME, United States) to generate *Prkaa1*^Lox/Lox^; Lysm^Cre/+^ (*Prkaa1*^Δ^ ^Mφ^) mice, and then these mice were further bred with apolipoprotein E-deficient mice (*Apoe*^–/–^ mice, Cat. No. 002052, the Jackson Laboratory, Bar Harbor, ME, United States) to generate *Apoe*^–/–^/*Prkaa1*^Δ^ ^Mφ^ and *Apoe*^–/–^/*Prkaa1*^WT^ mice. All mice were bred on the C57BL/6J background. In this study, both male and female deficient mice and their littermate controls were used for experiments. Mice were generally maintained on a 12:12-h of light: dark cycle in temperature-controlled cages and had free access to water and diet. All animal experiments were performed in accordance with the National Institutes of Health guidelines and approved by the IACUC (Institutional Animal Care & Use Committee) of Augusta University.

### Glucose and Insulin Tolerance Tests

Six-week-old *Prkaa1*^ΔMφ^ and *Prkaa1*^WT^ mice were fed a high-fat diet (HFD) (Cat. No. D12492, Research Diet, New Brunswick, NJ, United States) for 12 weeks. For glucose tolerance test (GTT), mice fed with HFD for 10 weeks were fasted for 6 h and administered D-glucose (1 g/kg; Cat. No. G8270, Sigma-Aldrich, St. Louis, MO, United States) by intraperitoneal (IP) injection. Insulin tolerance was assessed after 4 h fast by IP injection of insulin (1 U/kg; Cat. No. HI0219, Lilly, Indianapolis, IN, United States). Blood samples were collected from the tail vein and the blood glucose was measured with a glucometer (OneTouch UltraEasy, Johnson & Johnson, New Brunswick, NJ, United States) at the indicated time after injection.

### Evaluation of Energy Homeostasis

The volume of carbon dioxide production (VCO_2_), volume of oxygen consumption (VO_2_), energy expenditure, and food and drink intake were monitored individually in *Prkaa1*^ΔMφ^ and *Prkaa1*^WT^ mice fed the HFD for 12 weeks with Comprehensive Lab Animal Monitoring System (CLAMS) (Columbus Instruments, Columbus, OH, United States). The ratio of VCO_2_ to VO_2_ was calculated for the respiratory exchange ratio (RER). Body composition of fat and lean mass was determined by a nuclear magnetic resonance (NMR) system (MiniSpec LF90II TD-NMR Analyzer, Bruker, Billerica, MA, United States).

### Leukocyte Sorting From Adipose Tissue

Epididymal adipose tissue was excised from *Prkaa1*^ΔMφ^ and *Prkaa1*^WT^ mice and minced into small pieces, digested with collagenase type I (5 mg/ml, Cat. No. 4194, Worthington, Lakewood, NJ, United States) in DMEM (Cat. No. 11054020, Gibco, Waltham, MA, United States) for 30 min at 37°C with shaking. After centrifugation at 500 *g* for 5 min, the stromal vascular fraction (SVF) was resuspended in FACS buffer (0.5% FBS in PBS) and passed through a 70-μm cell strainer. The cells were incubated with FcR blocking reagent (Cat. No. 553142, BD Biosciences, San Jose, CA, United States) for 10 min at 4°C, then incubated with 7-AAD (Cat. No. 559925, BD Biosciences, San Jose, CA, United States), PE anti-mouse CD31 (4 μg/ml; Cat. No. 553373, BD Biosciences, San Jose, CA, United States) and APC anti-mouse CD45 (3 μg/ml; Cat. No. 558702, BD Biosciences, San Jose, CA, United States) for 30 min at 4°C. After washing, the cells were sorted using a FACS Caliber. The CD45^+^CD31^–^7-AAD^–^ population was collected for RT-PCR analysis.

### Atherosclerotic Lesion Analysis

*Apoe*^–/–^/*Prkaa1*^ΔMφ^ and *Apoe*^–/–^/*Prkaa1*^WT^ male and female mice at 7 weeks of age were fed a Western diet (Cat. No. TD88137, ENVIGO, Indianapolis, IN, United States) for 16 weeks. Mice were anesthetized and the blood samples were collected. After that, mouse hearts and aorta were dissected after perfusion with phosphate buffered saline (PBS) (Cat. No. BP665-1, Fisher Scientific, Pittsburgh, PA, United States) and 4% paraformaldehyde (PFA) (Cat. No. sc281692, Santa Cruz Biotechnology, Dallas, TX, United States), respectively. Tissues were then fixed with 4% PFA overnight. For the preparation of aortas, the periadventitial fat and connective tissue were removed, and atherosclerotic lesions on the aortas were stained with 2% Oil Red O (Cat. No. O0625, Sigma-Aldrich, Louis, MO, United States). The size of atherosclerotic lesions was evaluated by a quantification of *en face* Oil Red O staining with Image-Pro Plus software (Media Cybernetics, Bethesda, MD, United States) in a blinded manner.

For the preparation of aortic sinus, one-third of the heart with aortic root was cut transversally and embedded in optimum cutting temperature (OCT) compound (Cat. No. 23-730-571, Fisher Scientific, Waltham, MA, United States) and further processed to 5-μm-thick frozen sections of aortic sinus for Hematoxylin and Eosin (HE) and Oil Red O staining. The size of necrotic cores and lipid deposition in the aortic sinus were quantified with Image-Pro Plus software in a blinded manner. Nine sections were quantified for each mouse in each group.

### Histology

To characterize the necrotic areas in atherosclerotic lesions, HE staining was performed on 5 μm-thick sections of aortic sinus with Hematoxylin (Cat. No. 22050111, Thermo Scientific, Waltham, MA, United States) and Eosin (Cat. No. 22050110, Thermo Scientific, Waltham, MA, United States). For analysis of collagen and fibrosis, Masson trichrome staining was performed on 5 μm-thick sections of aortic sinus with Masson’s Trichrome Stain Kit (Cat. No. KTMTR, American MasterTech, Lodi, CA, United States) according to the manufacturer’s instruction. For Mac-2 immunohistochemistry staining on mouse adipose, sections of adipose were quenched with 3% hydrogen peroxide and blocked with 10% goat serum. Sections were then incubated with anti-Mac-2 (3 μg/ml, Cat. No. ACL8942F, Accurate Chemical & Scientific, Westbury, NY, United States) at 4°C overnight. Biotinylated secondary antibodies were incubated and VECTASTAIN^®^ ABC kit (Cat. No. PK-6100, Vector Labs, Burlingame, CA, United States) were applied according to the manufacturer’s instructions. Necrotic area and collagen content and Mac-2 staining area were quantified with Image-Pro Plus software.

### Immunofluorescence Analysis

Frozen sections were fixed with 4% PFA and permeabilized with PBS containing 0.5% Triton X-100. Paraffin sections were heated in citric acid buffer (10 mM, PH6.0) at 98°C for 10 min for antigen retrieval. Tissues were blocked with 10% normal goat serum (Cat. No. 50062Z, Thermo Fisher Scientific, Waltham, MA, United States) at room temperature for 1 h and incubated with primary antibodies, anti-Mac2 (Cat. No. ACL8942F, Accurate Chemical & Scientific, Westbury, NY, United States), anti-Prkaa1 (Cat. No. GTX112998, GeneTex, Irvine, CA, United States), anti-P-Prka (Cat. No. GTX52341, GeneTex, Irvine, CA, United States), anti-CD36 (Cat. No. Ab80080, Abcam, Cambridge, United Kingdom), anti-Pfkfb3 (Cat. No. 13763-1-AP, Proteintech, Rosemont, IL, United States), anti-Slc2a1 (Cat. No. Ab115730, Abcam, Cambridge, United Kingdom), anti-F4/80 (Cat. No. Ab6640, Abcam, Cambridge, United Kingdom), anti-CD68 (Cat. No. MA5-13324, Thermo Scientific, Waltham, MA, United States) at 4°C overnight. Sections were then incubated with AlexaFluor conjugated secondary antibodies (1:250, Invitrogen, Grand Island, NY, United States) or TUNEL mixture for 1 h according to the manufacturer’s instructions. Sections were stained with DAPI (1 μg/mL, Thermo Fisher Scientific) for 5 min at room temperature and mounted. Images were obtained using an upright confocal microscope (Zeiss 780; Carl Zeiss).

### Leukocyte Recruitment in the Mouse Cremaster Microcirculation

To perform the leukocyte recruitment analysis, the cremaster muscle model of TNF-α-induced inflammation was used. Each group included four *Prkaa1*^ΔMφ^ and *Prkaa1*^WT^ male mice at 8–12 weeks old. Briefly, mice were injected IP with 10 μg/kg recombinant murine TNF-α (Cat. No. 410-MT-010, R&D Systems, Minneapolis, MN, United States). 4 h later, mice were anesthetized with ketamine (125 mg/kg) and xylazine (12.5 mg/kg). Cremaster muscle was exteriorized from the scrotum and spread on the cover glass and superfused with warmed (35–37°C) saline to keep it wet. Movies were obtained with intravital microscopy (Axioskop; Carl Zeiss, Goettingen, Germany) with a digital camera (AxioCam MRm, Carl Zeiss) and analyzed using digital video software (ZEN 2012, Carl Zeiss). For each cremaster, 3–4 different postcapillary venules were examined and 3 different segments were recorded for each venule. In total, 36–48 video clips were obtained and analyzed for each group. The number of cells rolling through a perpendicular line to the vessel axis per minute was measured as rolling flux. Leukocyte adhesion was defined as leukocyte adhesion to endothelium more than 30 s and measured as cell numbers per surface area. Surface area was calculated for each vessel as S = π^∗^d^∗^lV where d is the diameter and lV is the length of the vessel.

### Plasma Cholesterol, Triglyceride, Glucose, and Blood Leukocyte Analysis

Blood was drawn from *Apoe*^–/–^/*Prkaa1*^WT^ and *Apoe*^–/–^/*Prkaa1*^ΔMφ^ mice that were fasted for 12–14 h following 16 weeks of being fed a Western diet. The subsequent plasma samples were assayed enzymatically with the respective reagents (Cat. No. TR15421, TR13421, TR22421, Thermo Scientific) for measurement of glucose, cholesterol, and triglycerides. Leukocyte numbers in blood were counted with an automated blood cell counter (Hemavet 850FS, CDC Technologies, Oxford, CT, United States).

### Bone Marrow Derived Macrophage (BMDM) Culture and Treatments

*Prkaa1*^WT^ and *Prkaa1*^ΔMφ^ mice at 8 weeks of age were euthanized. Femurs and tibias were isolated and transected. Bone marrow cells were obtained by flushing femurs and tibias with RPMI 1640 medium (Cat. No. SH30809.01B, HyClone, Logan, UT, United States). The cell suspension was pipetted repeatedly to obtain a single cell suspension, which was then filtered with a 70-μm cell strainer and centrifuged at 2000 rpm for 5 min. The acquired cells were plated at a density of 2 × 10^6^/mL and cultured in RPMI 1640 medium supplemented with 10% FBS, 20% L929-conditioned medium, and 1% penicillin-streptomycin. Cells were incubated at 37°C, 5% CO_2_ with medium changes at days 3, 5, and 7. After 7 days, the bone marrow cells were differentiated into adherent macrophages, which were used to perform experiments.

### Extracellular Acidification Rate (ECAR) Measurement

Extracellular acidification rate (ECAR) of BMDMs was analyzed with an XF96 Extracellular Flux Analyzer (Seahorse Bioscience, North Billerica, MA, United States). Wild-type (WT) and *Prkaa1-*deficient BMDMs were first plated onto Seahorse XF96 cell culture plates at a concentration of 2.0 × 10^4^ per well, and incubated with RPMI 1640 medium at 37°C overnight. The next day, the culture medium was changed to XF base Medium (Seahorse Bioscience) supplemented with 2 mM glutamine, and the cells were incubated in a non-CO_2_ incubator at 37°C for 1 h. Cells were then assayed with XFe96 extracellular flux analyzer. Chemicals were used in the test at the following concentration: glucose (10 mM), oligomycin (1 μM), and 2-DG (50 mM).

### Fatty Acid Oxidation Rate Measurement

Fatty acid oxidation was measured by XFe24 Seahorse metabolic flux analyzer (Seahorse Bioscience, North Billerica, MA, United States). WT and *Prkaa1-*deficient BMDMs were first seeded onto Seahorse XF24 culture cell plates at a concentration of 1.0 × 10^5^ per well, and incubated with RPMI 1640 medium at 37°C overnight. The next day, the medium was replaced with substrate-limited medium (DMEM supplemented with 0.5 mM Glucose, 1 mM GlutaMAX, 0.5 mM carnitine and 1% FBS) for 24 h. Then cells were incubated with FAO assay medium (KHB supplemented with 2.5 mM glucose, 0.5 mM carnitine, and 5 mM HEPES, pH 7.0) for 45 min in a non-CO_2_ incubator at 37°C. XF Palmitate-BSA FAO Substrate or BSA (Cat. No. 102720-100, Agilent, Santa Clara, CA, United States) was added to the appropriate wells prior to starting the assay. Plates were then loaded onto an XFe24 extracellular flux analyzer for XF Cell Mito Stress Test. Compounds were used in the test at the following concentrations: oligomycin (2 μM), FCCP (2 μM), rotenone (1 μM), and antimycin A (1 μM). Fatty acid oxidation was calculated by subtracting oxygen consumption rate (OCR) with palmitate-BSA by OCR with BSA. Spare respiratory capacity was calculated by subtracting baseline OCR from maximum OCR after FCCP injection.

### Lactate Measurement

The lactate levels of WT and *Prkaa1*-deficient BMDMs were determined with the Lactate Assay Kit (Cat. No. MAK064, Sigma, Louis, MO, United States) according to the manufacturer’s instructions. Briefly, cells were homogenized with lactate assay buffer and centrifuged at 13,000 *g* for 10 min. Samples were deproteinized with 10 kDa MWCO spin filter, and the soluble fraction was used directly to measure the intracellular lactate. The cell culture medium was used for measurement of the extracellular lactate level.

### F-2,6-P2 Level Assay

The intracellular F-2, 6-P2 level was measured with the previously described method (Van Schaftingen et al., 1982). WT and *Prkaa1*-deficient BMDMs were homogenized with 0.05M NaOH and inactivated at 80°C for 5 min. The samples were then centrifuged and neutralized with acetic acid. The mixture was centrifuged and the supernatants were collected for measurement. The levels of F-2,6-P2 were measured by a PPi-PFK-based kinetic reaction assay as shown in the table below (Table 1). The kinetic reaction was monitored at 340 nm for 30 min with Synergy H1 Hybrid Reader (BioTek, Winooski, VT, United States). The relative F2,6-P2 levels were normalized by protein concentration.

**TABLE 1:** Assay reaction for F2,6-P2 level.

Substrate	Initial concentration	Work concentration
Tris-HCl (pH7.5)	50 mM	50 mM
NADH	20 mM	0.2 mM
DTT	1 M	5 mM
F6P	200 mM	1 mM
MgCl2	1 M	2 mM
Aldolase	700 U/ml	0.7 U/ml
GDH	450 U/ml	0.45 U/ml
TIM	1200 U/ml	0.6 U/ml
PPi-Na	25 mM	0.5 mM
PPi-PFK		10 μg/assay

### Apoptosis Analysis

Apoptosis analysis were performed with fluorescein isothiocyanate (FITC) Annexin V Apoptosis Detection Kit I (Cat. No. 556547, BD Biosciences) according to the manufacturer’s protocol. Briefly, WT and *Prkaa1*-deficient BMDMs were seeded into 6-well plates at a density of 2 × 10^6^ cells/ml. After incubation for 24 h, cells were detached with 0.25% trypsin and incubated with 5 μl propidium iodide (PI) and 5 μl FITC Annexin V in 100 μl of 1 × binding buffer at room temperature for 15 min in the dark. The samples were analyzed by FACSCalibur flow cytometer (FACSCantor II, BD Biosciences) within 1 h. Apoptotic-positive cells were quantified with FlowJo.

For terminal transferase deoxytidyl uridine end labeling (TUNEL) staining, WT and *Prkaa1*-deficient BMDMs were seeded on glass coverslips at a density of 2 × 10^6^ cells/ml. After incubation for 24 h, cells were stained with *In Situ* Cell Death Detection Kit (Cat. No. 12156792910, Roche) according to the manufacturer’s protocol. Briefly, cells were washed with PBS twice, fixed with 4% PFA for 15 min and permeabilized with PBS containing 0.5% Triton X-100 for 20 min at room temperature. Cells were then incubated with the TUNEL mixture containing TMR-dUTP and terminal deoxynucleotidyl transferase for 1 h at 37°C in the dark. Nuclei were stained with DAPI before mounting. Images were obtained using an upright confocal microscope (Zeiss 780, Carl Zeiss). The number of TUNEL-positive cells was counted in seven fields per slide.

### Chemotaxis Assays

Chemotaxis assay of WT and *Prkaa1*-deficient BMDMs was performed in a transwell chamber equipped with a 6.5-mm-diameter polycarbonate filter with 8-μm pore inserts (Cat. No.353097, Corning, NY, United States). Briefly, RPMI 1640 medium containing 0.5% FBS with or without MCP-1 (50 ng/ml, Cat. No.479-JE, R&D Systems Inc., Minneapolis, MN, United States) was placed in the lower wells. 2 × 10^5^ BMDMs suspended in RPMI 1640 containing 0.5% FBS were placed in the inserts. The chambers were incubated at 37°C for 8 h. Non-migrated cells on the upper side of inserts were removed with a cotton tip, and migrated cells on the lower side were fixed and stained with crystal violet. BMDMs in 5–7 random fields were counted for each transwell.

### Western-Blot Analysis

Wild-type and *Prkaa1*-deficient BMDMs were lysed with RIPA lysis buffer (Sigma) supplemented with 1% phosphatase inhibitors and 1% protease (Roche) for 10 min on ice. After centrifugation of cell lysates at 4°C for 5 min, samples were assayed for protein concentrations by the BCA assay and then separated with SDS-PAGE gel with 10–20 μg protein per lane. Western-blot analysis was performed with primary antibodies against Prkaa1 (1:1000, Cat. No. ab110036, Abcam), Slc2a1 (1:5000, Cat. No. ab115730, Abcam), Pfkfb3 (1:1000, Cat. No. ab181681, Abcam), and β-actin (1:2000, Cat. No. sc47778, Santa Cruz Biotechnology). Images were obtained with the CheminDoc MP System (Bio-Rad) and quantified with Image J software. All protein levels were normalized with β-actin level.

### Real-Time PCR Analysis

Total RNA of WT and *Prkaa1*-deficient BMDMs were extracted using Trizol Reagent (Cat. No. 15596018, Invitrogen, Grand Island, NY, United States). 0.5–1 μg total RNA was used for reverse transcription reaction with iScript cDNA synthesis kit (Cat. No. 170-8891, Bio Rad, Hercules, CA, United States). Real-Time PCR was performed in a StepOne Plus system (Applied Biosystems) with Power SYBR Green PCR Master Mix (Cat. No. 4367659, Life Technologies) according to the manufacturer’s protocol. Gene-specific primers used in this study are listed in Table 2. Relative gene expression was calculated with the efficiency-corrected 2^–△△^^CT^ method by using mouse Rplp0 RNA as the internal control.

**TABLE 2:** Sequences of primers used in real-time PCR analysis.

Gene name	Forward primer (5′-3′)	Reverse primer (5’-3’)
*Rplp0*	GGCCCTGCACTCTCGCTTTC	TGCCAGGACGCGCTTGT
*Scl2a1*	GCAGTTCGGCTATAACACTGG	GCGGTGGTTCCATGTTTGATTG
*Pfkfb3*	GATCTGGGTGCCCGTCGATC ACCG	CAGTTGAGGTAGCGAGTCAGCTTC
*Hk1*	AACGGCCTCCGTCAAGATG	GCCGAGATCCAGTGCAATG
*Pkm2*	AGGATGCCGTGCTGAATG	TAGAAGAGGGGCTCCAGAGG
*ldha*	CCAAAGACTACTGTGTAACT GCGA	TGGACTGTACTTGACAATG TTGG
*Eno1*	TGCGTCCACTGGCATCTAC	CAGAGCAGGCGCAATAGTTTTA
*Gpi*	CTCAAGCTGCGCGAACTTTTT	GGTTCTTGGAGTAGTCCACCAG
*Cd36*	TGCTGGAGCTGTTATTGGTG	TCTTTGATGTGCAAAACCCA
*Cpt1a*	GTCGCTTCTTCAAGGTCTGG	AAGAAAGCAGCACGTTCGAT
*Fabp4*	AAGAAGTGGGAGTGGGCTTT	TCGACTTTCCATCCCACTTC

### Statistical Analysis

Statistical analysis was conducted with GraphPad Prism (La Jolla, CA, United States). The significance of the data was analyzed using unpaired Student’s *t*-test between two groups. Multiple comparisons were performed with one-way ANOVA analysis followed by Bonferroni’s *post hoc* tests. Statistical significance was defined as follows: ^∗^*p* < 0.05, ^∗∗^*p* < 0.01, ^∗∗∗^*p* < 0.001. Data are represented as mean ± SEM. All biological experiments were repeated at least three times with independent cell cultures or individual animals (biological replications).

## Results

### *Prkaa1* Expression Is Increased in Adipose Leukocytes of HFD-Fed Mice

**FIGURE 1:**
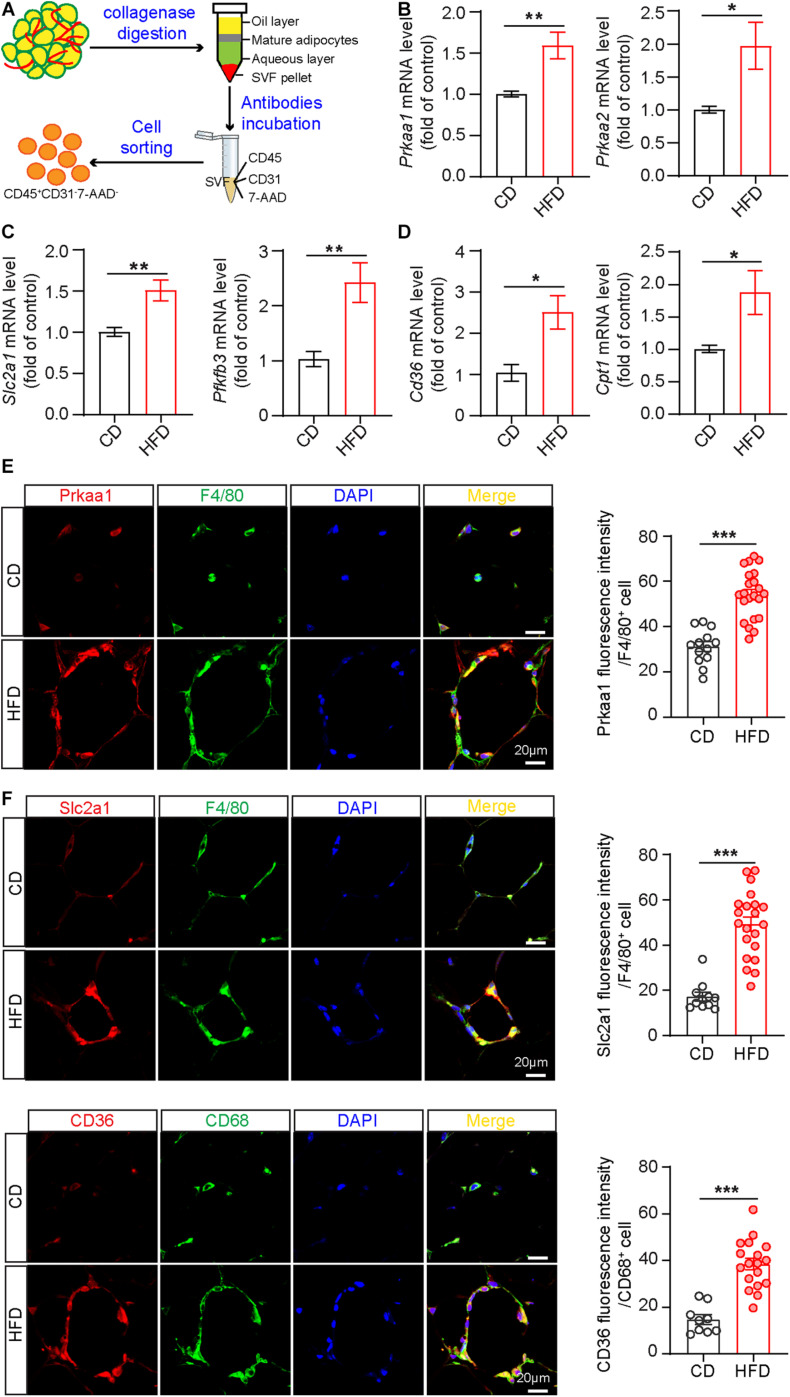
Prkaa1 upregulation is associated with macrophage metabolism in mice fed a high-fat diet. **(A)** Schema illustrating leukocyte sorting from adipose tissues by CD45-positive staining. **(B)** Real-time PCR analysis and quantification of mRNA levels of *Prkaa1* and *Prkaa2* in leukocytes sorted from stromal vascular cells in HFD- and CD-fed mice. *n* = 8. **(C,D)** Real-time PCR analysis and quantification of mRNA levels of *Slc2a1*, *Pfkfb3*, *Cd36*, and *Cpt1* in leukocytes sorted from stromal vascular cells in HFD- and CD-fed mice. *n* = 8. **(E,F)** Representative images and quantification data of Prkaa1, Slc2a1, and CD36 co-staining with F4/80 or CD68 (macrophage markers) in adipose tissues from HFD- and CD-fed mice. *n* = 4, Scale bars, 20 μm. All data were expressed as mean ± SEM. Statistical significance was determined by unpaired Student’s *t*-test. *p < 0.05 was considered significant, **p < 0.01, ***p < 0.001.

Obesity is a chronic, low-grade inflammation with progressive immune cell infiltration into adipose tissue (Bai and Sun, 2015). Many circulating leukocytes, especially monocytes, recruit to adipose tissue to enhance the inflammatory response, accelerating adipocyte necrosis and insulin resistance (Catalan et al., 2013). To explore Prka expression in infiltrating leukocytes in the context of metabolic diseases, we performed fluorescence-activated cell sorting (FACS) to isolate leukocytes from adipose tissues. Briefly, we digested adipose tissue with collagenase I to obtain SVFs, followed by incubation of SVFs with antibodies against CD31 (endothelial cell marker), CD45 (leukocyte marker) and 7-AAD (dead cell marker), and eventually harvested cells labeled as CD31^–^CD45^+^ 7-AAD^–^ from the cell suspension (Figure 1A and Supplementary Figure 1). Next, the sorted leukocytes were subjected to Real-time PCR analysis for the mRNA expression of *Prkaa1* and *Prkaa2*. The results showed that the mRNA levels of *Prkaa1* and *Prkaa2* were much higher in leukocytes from adipose tissues of mice fed HFD for 8 weeks than mice fed chow diet (CD) (Figure 1B). Prkaa1 is the major isoform of Prkaa in leukocytes (Zhang et al., 2017), and our recent study shows a critical role of Prkaa1 in metabolic regulation in vascular cells (Yang et al., 2018). We analyzed expression of metabolic genes in sorted cells with qPCR. The mRNA levels of *Slc2a1*, a glucose transporter, and 6-phosphofructo-2-kinase/fructose-2, 6-bisphosphatase isoform 3 (*Pfkfb3*), a critical glycolytic regulatory enzyme (activator), were much higher in adipose leukocytes of HFD-fed mice than those of CD-fed mice (Figure 1C). Additionally, the mRNA levels of *Cd36*, a transporter for free fatty acids and *Cpt1*, a mitochondrial enzyme important for fatty acid oxygenation, were also increased in adipose leukocytes of HFD-fed mice compared with those of chow diet-fed mice (Figure 1D). Since macrophages are the predominate leukocytes infiltrating in adipose, we examined the expression of Prkaa1 and some of the above metabolic molecules in F4/80-positive cells by immunostaining adipose sections with the relevant antibodies. The levels of Prkaa1, pPrkaa1, Slc2a1, Pfkfb3 and CD36 were much higher in HFD-fed mice than CD-fed mice (Figures 1E,F and Supplementary Figure 2). Taken together, the data suggest that leukocytes infiltrated into adipose tissue, especially monocytes/macrophages, exhibit increased levels of Prkaa1, and the latter is associated with increased expression of molecules important for glucose and lipid metabolism.

### Prkaa1 Regulates Macrophage Glycolysis

**FIGURE 2:**
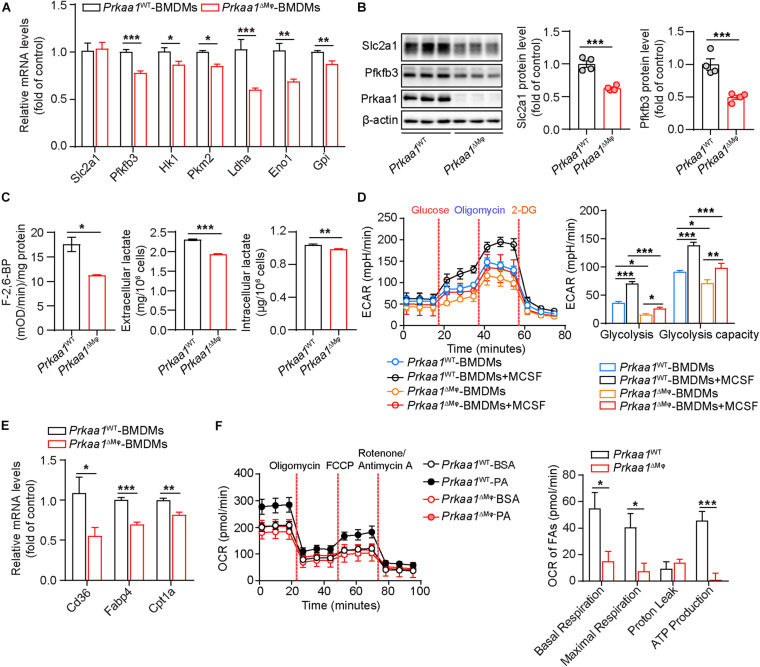
Prkaa1 regulates macrophage glycolysis and fatty acid oxidation. **(A)** Real time-PCR analysis and quantification of mRNA levels of *Slc2a1*, *Pfkfb3*, *Hk1*, *Pkm2*, *Ldha*, *Eno1*, and *Gpi* in BMDMs cultured from *Prkaa1*^WT^ and *Prkaa1*^ΔMφ^ mice. *n* = 4. **(B)** Western-blot analysis and quantification of protein levels of Slc2a1, Pfkfb3, and Prkaa1 in BMDMs cultured from *Prkaa1*^WT^ and *Prkaa1*^ΔMφ^ mice. *n* = 4. **(C)** Measurement of F-2,6-BP, intracellular and extracellular lactate in BMDMs cultured from *Prkaa1*^WT^ and *Prkaa1*^ΔMφ^ mice. *n* = 6. **(D)** ECAR profile showing glycolytic function and quantification of glycolytic function parameters in BMDMs cultured from *Prkaa1*^WT^ and *Prkaa1*^ΔMφ^ mice under control and MCSF (20 ng/mL, 12 h) treatment. Dash lines indicate the time of addition of glucose (10 mM), oligomycin (1 μM), and 2-DG (50 mM). *n* = 12 for each treatment group, repeated four times. **(E)** Real time-PCR analysis and quantification of mRNA levels of *Cd36*, *Fabp4*, and *Cpt1a* in BMDMs cultured from *Prkaa1*^WT^ and *Prkaa1*^ΔMφ^ mice. *n* = 4. **(F)** OCR profile showing fatty acid oxidation and quantification of function parameters in BMDMs cultured from *Prkaa1*^WT^ and *Prkaa1*^ΔMφ^ mice under BSA-palmitate treatment. Etomoxir (Eto, 40 μM) was added to some wells 15 min prior to the assay. *n* = 12 for each treatment group, repeated four times. All data are expressed as mean ± SEM. Statistical significance was determined by unpaired Student’s *t*-test and one-way ANOVA followed by Bonferroni test. *p < 0.05 was considered significant, **p < 0.01, and ***p < 0.001.

Since macrophages are the major population of infiltrated leukocytes in adipose, we used cultured BMDMs to examine the causal effect of *Prkaa1* on macrophage metabolism. BMDMs were cultured with bone marrow cells from *Prkaa1*^WT^ and *Prkaa1*^ΔMφ^ mice (Supplementary Figures 3A,B). BMDMs cultured with bone marrow cells of *Prkaa1*^ΔMφ^ mice barely expressed Prkaa1 at the protein level compared to BMDMs of control *Prkaa1*^WT^ mice (Supplementary Figure 3C), indicating that *Prkaa1* was successfully knocked out in myeloid cells. With qPCR, the major genes important for glycolysis were examined, and the mRNA levels of glycolytic genes, including *Pfkfb3, Hk1, Pkm2, Ldha, Eno1*, and *Gpi*, were significantly reduced in *Prkaa1*-deficient BMDMs compared with those of *Prkaa1* WT BMDMs (Figure 2A). Expression of Slc2a1 and Pfkfb3 were examined with Western blotting. The protein levels of these molecules were much lower in *Prkaa1*-deficient BMDMs than WT BMDMs (Figure 2B). Posttranscriptional regulation of Slc2a1 by Prkaa1 may occur since the decreased expression of Prkaa1 at the protein, but not mRNA, level was observed in *Prkaa1*-deficient BMDMs. Consistent with the decreased expression of Pfkfb3, the level of fructose-2,6-biphosphate [(the product of Pfkfb3, and the most potent allosteric activator of 6-phosphofructo-1-kinase (PFK1)] was also decreased in *Prkaa1*-deficient BMDMs (Figure 2C). The level of lactate, the end product of glycolysis, was also reduced in *Prkaa1-*deficient BMDMs compared with that in control cells (Figure 2C). Importantly, we assessed glycolytic metabolism in *Prkaa1*-deficient BMDMs using Seahorse Extracellular Flux analysis via measurement of the ECAR. As shown in Figure 2D, the glycolytic metabolism was increased in BMDMs stimulated with MCSF. In contrast, *Prkaa1*-deficient BMDMs exhibited significantly reduced basic glycolysis and glycolytic capacity compared with control cells either in the presence or absence of MCSF. Altogether, these results indicate that PRKAA1/*Prkaa1* is an endogenous regulator of glycolysis in macrophages.

We also evaluated fatty acid oxidation (FAO) in these BMDMs. The mRNA levels of FAO-associated genes, including *Cd36, Fabp4*, and *Cpt1a*, were significantly decreased in *Prkaa1*-deficient BMDMs compared with those in control BMDMs (Figure 2E). We next performed the Seahorse assay to determine the OCR in the presence of palmitate. As shown in Figure 2F, FAO was dramatically decreased in *Prkaa1*-deficient BMDMs compared with control cells. These results collectively suggest that Prkaa1 regulates FAO in macrophages.

### Prkaa1 Deficiency Reduces Leukocyte Recruitment and Macrophage Viability

**FIGURE 3:**
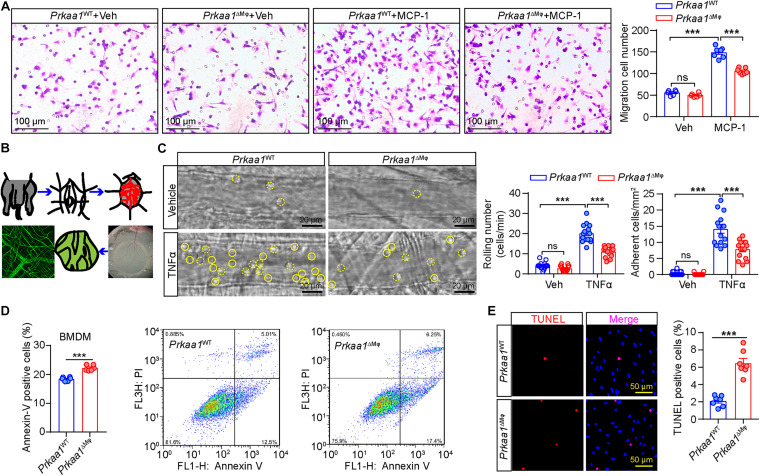
Prkaa1-mediated metabolism is required for leukocyte recruitment and macrophage viability. **(A)** Representative images and quantification of MCP-1-induced migration between BMDMs cultured from *Prkaa1*^WT^ and *Prkaa1*^ΔMφ^ mice. *n* = 7. Scale bars, 100 μm. **(B)** Schematic illustration of *en face* staining of postcapillary venules of mouse cremaster muscle. **(C)** Representative images of leukocyte rolling and adhesion on the endothelium of postcapillary venules in the cremaster muscles of *Prkaa1*^ΔMφ^ and *Prkaa1*^WT^ mice with TNFα or vehicle treatment for 4 h. Rolling and adherent cells are quantified and indicated with dotted and solid circle line, respectively (scale bar, 20 μm). **(D)** Quantification data and representative images of flow cytometry analysis of Annexin V staining in BMDMs cultured from *Prkaa1*^WT^ and *Prkaa1*^ΔMφ^ mice. *n* = 6. **(E)** Representative images and quantification data of TUNEL staining in BMDMs cultured from *Prkaa1*^WT^ and *Prkaa1*^ΔMφ^ mice. *n* = 10, Scale bars, 50 μm. All data are expressed as mean ± SEM. Statistical significance was determined by unpaired Student’s *t*-test. ^∗∗∗^*p* < 0.001.

Glycolysis and FAO are important for leukocyte recruitment and survival. Therefore, we examined whether the decreased metabolism in PRKAA1/*Prkaa1*-deficient leukocytes affects leukocyte recruitment and viability. We first compared the migration capability of WT and *Prkaa1*-deficient BMDMs. In a transwell migration assay, WT macrophages exhibited robust migration to medium supplemented with MCP-1. This ability was markedly decreased for *Prkaa1*-deficient BMDMs (Figure 3A). To determine the functional consequences of Prkaa1 deficiency in leukocyte recruitment *in vivo*, *Prkaa1*^ΔMφ^ and control *Prkaa1*^WT^ mice were injected with TNF-α (10 μg/ml) IP to induce vascular inflammation *in vivo*. Four hours after TNF-α treatment, leukocyte rolling and adhesion in the endothelium of postcapillary venules of mouse cremaster muscle were observed with intravital microscopy (Figure 3B). Mild trauma caused by the exteriorization of the cremaster muscle led to fast leukocyte rolling. In *Prkaa1*^ΔMφ^ mice, the number of fast rolling leukocytes were comparable to that in *Prkaa1*^WT^ mice (Figure 3C). TNF-α treatment stimulated the expression of adhesion molecules on the endothelium and induced slow rolling and adhesion of leukocytes in mice. The numbers of slow rolling and adhesion of leukocyte were reduced by 40–50% in *Prkaa1*^ΔMφ^ mice compared with those in control mice (Figure 3C). These results indicate compromised recruitment ability of *Prkaa1*-deficient leukocytes in mice. Taken together, these *in vitro* and *in vivo* findings support that Prkaa1-mediated metabolism is required for leukocyte recruitment and macrophage viability.

We also tested whether Prkaa1-mediated metabolism is important for viability of BMDMs. Flow cytometry analysis showed the percentage of Annexin V-positive BMDMs was much higher in *Prkaa1*-deficient BMDMs than WT cells (Figure 3D). Consistent results were obtained in BMDMs stained with TUNEL (Figure 3E). In mimicking the *in vivo* diabetic condition, we performed the above assays using BMDMs treated with high glucose (25 mM). The percentage of Annexin V-positive BMDMs was also increased for *Prkaa1*-deficient cells compared with that for WT cells (Supplementary Figure 4). These results reveal increased rates of apoptosis in the *Prkaa1*-deficient myeloid cells.

### Myeloid *Prkaa1* Deficiency Protects Mice From HFD-Induced Insulin Resistance

**FIGURE 4:**
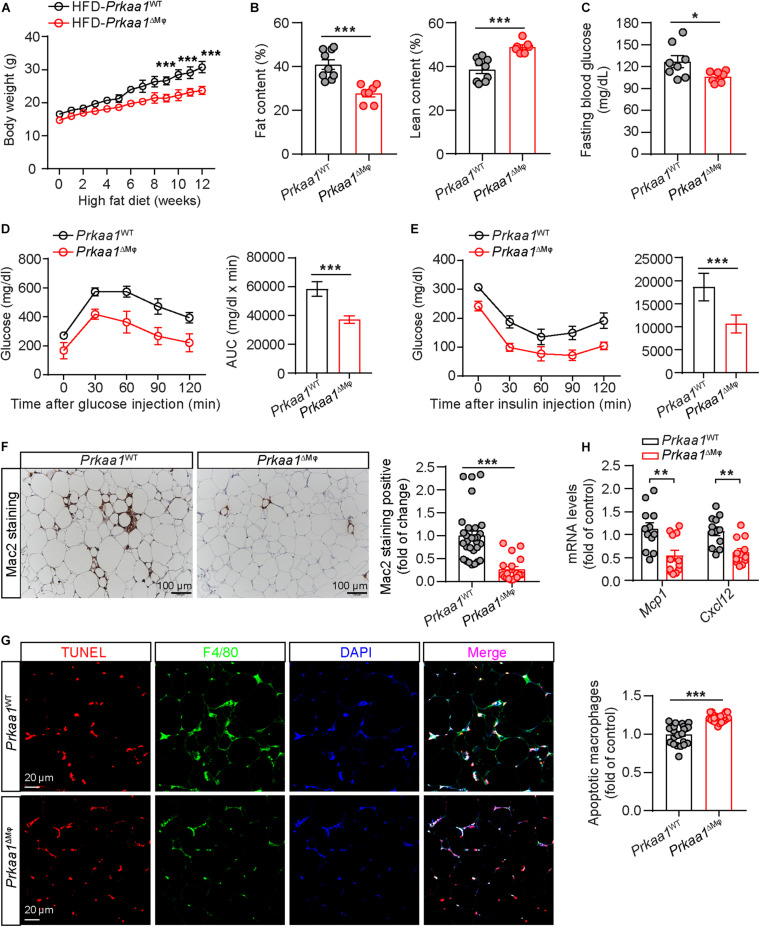
Myeloid *Prkaa1* deficiency protects mice from HFD-induced insulin resistance. **(A)** Body weight of *Prkaa1*^WT^ and *Prkaa1*^ΔMφ^ male mice during 12 weeks of HFD feeding. *n* = 9. **(B)** Fat and lean content of *Prkaa1*^WT^ and *Prkaa1*^ΔMφ^ male mice after being fed HFD for 12 weeks. *n* = 9. **(C)** Fasting blood glucose levels of *Prkaa1*^WT^ and *Prkaa1*^ΔMφ^ male mice after being fed HFD for 12 weeks. *n* = 8. **(D)** Blood glucose levels (left) and AUC (area under the curve, right) during GTT (glucose tolerance test) in *Prkaa1*^WT^ and *Prkaa1*^ΔMφ^ male mice after being fed HFD for 10 weeks. *n* = 6. Mice were fasted for 16 h and injected with glucose (2 g/kg IP). **(E)** Blood glucose levels (left) and AUC (right) during ITT (insulin tolerance test) in *Prkaa1*^WT^ and *Prkaa1*^ΔMφ^ male mice after being fed HFD for 11 weeks. *n* = 6. Mice were fasted for 4 h and injected with insulin (0.75 unit/kg body weight through IP injection). **(F)** Representative images and quantification of Mac2 (macrophage marker) staining in adipose tissues from *Prkaa1*^WT^ and *Prkaa1*^ΔMφ^ male mice after being fed HFD for 12 weeks. *n* = 5 mice/group, 5 areas/mice quantified. Scale bar, 100 μm. **(G)** Representative images and quantification of apoptotic macrophages (TUNEL and F4/80 double-positive cells) in adipose tissues from *Prkaa1*^WT^ and *Prkaa1*^ΔMφ^ male mice after being fed HFD for 12 weeks. *n* = 5 mice/group, 5 areas/mice quantified. Scale bar, 20 μm. **(H)** Quantitative RT-PCR analysis of the mRNA level of *Mcp1* and *Cxcl12* in adipose tissue from *Prkaa1*^WT^ and *Prkaa1*^ΔMφ^ male mice after being fed HFD for 12 weeks. *n* = 12 mice per group. All data are expressed as mean ± SEM. Statistical significance was determined by unpaired Student’s *t*-test. **p < 0.01, ***p < 0.001.

Leukocytes, including myeloid cells, participate in the development of HFD-induced metabolic syndrome (Weinstock et al., 2020). We examined whether *Prkaa1* deficiency-mediated decreased leukocyte recruitment affects diet-induced metabolic syndrome. After being fed of HFD for 12 weeks, *Prkaa1*^ΔMφ^ mice displayed resistance to HFD-induced body-weight gain compared with that of *Prkaa1*^WT^ mice (Figure 4A). Meanwhile, body composition analysis after feeding of HFD showed that *Prkaa1*^ΔMφ^ mice had a lower body fat content and correspondingly a higher percentage of lean mass than *Prkaa1*^WT^ mice (Figure 4B). Of note, the fasting blood glucose level was significantly reduced in *Prkaa1*^ΔMφ^ mice compared with that in *Prkaa1*^WT^ mice under the HFD condition (Figure 4C). Consistent with these results, *Prkaa1*^ΔMφ^ mice exhibited improved glucose clearance in GTTs, as well as improved insulin sensitivity in insulin tolerance tests (ITTs) compared with *Prkaa1*^WT^ mice (Figures 4D,E). Furthermore, Mac2 staining on adipose sections showed a significant reduction in macrophage infiltration into adipose of *Prkaa1*^ΔMφ^ mice compared with that of *Prkaa1*^WT^ mice (Figure 4F). Accordingly, lower levels of chemokines including Mcp-1 and Cxcl12 was also observed in adipose of *Prkaa1*^ΔMφ^ mice compared to *Prkaa1*^WT^ mice under the HFD condition (Figure 4H). Additionally, TUNEL-staining showed that the percentage of apoptotic cells among F4/80-positive macrophages was much higher in adipose of *Prkaa1*^ΔMφ^ mice than *Prkaa1*^WT^ mice (Figure 4G). These results suggest that the low number of myeloid cells due to decreased recruitment and increased apoptosis in adipose of *Prkaa1*^ΔMφ^ mice reduces HFD-induced metabolic syndrome.

**FIGURE 5:**
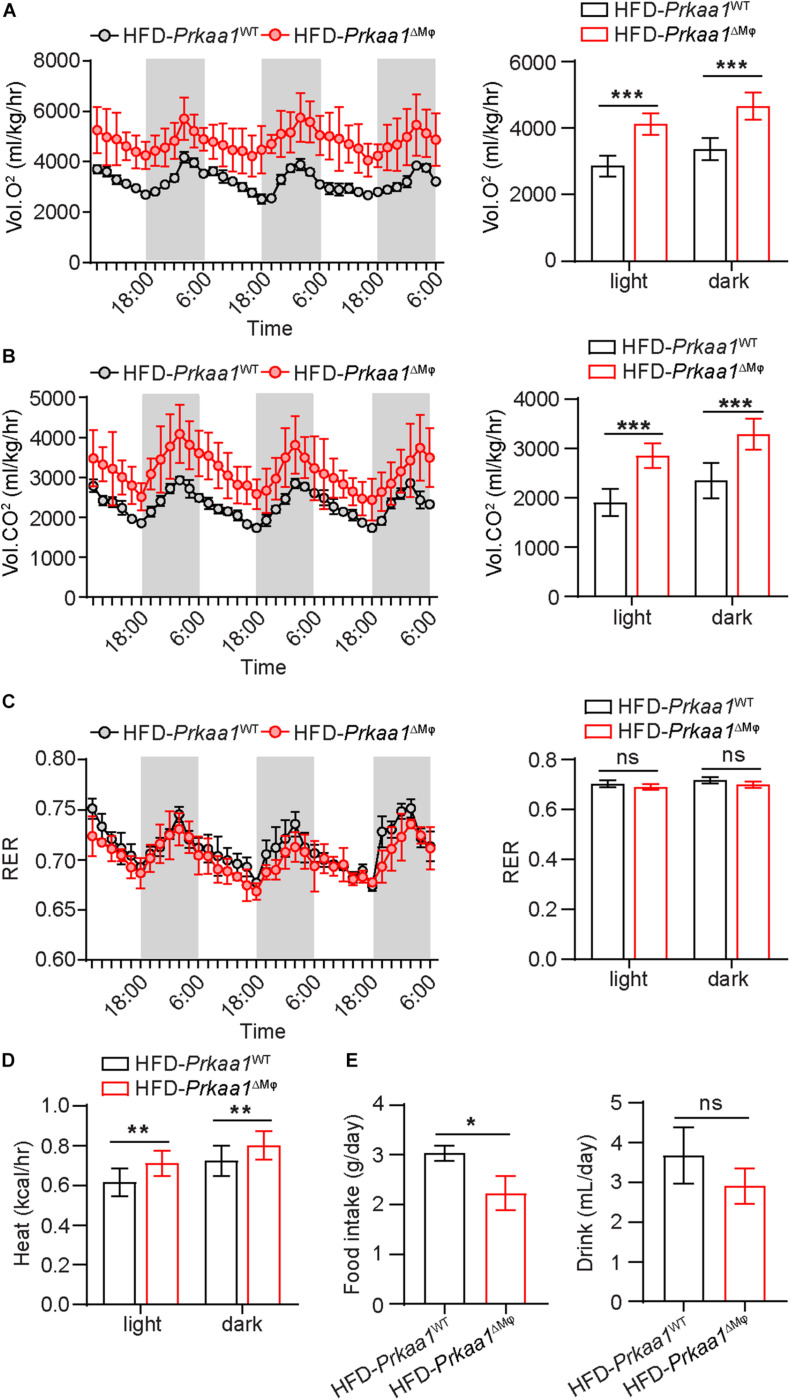
Myeloid *Prkaa1* deficiency increases energy expenditure in response to HFD. **(A)** Oxygen consumption (VO_2_) of *Prkaa1*^WT^ and *Prkaa1*^ΔMφ^ male mice during 12-h light and dark cycles recorded on the second day after acclimatization. **(B)** Carbon dioxide consumption (VCO_2_) of *Prkaa1*^WT^ and *Prkaa1*^ΔMφ^ male mice during 12-h light and dark cycles recorded on the second day after acclimatization. **(C)** Respiratory exchange ratio (RER) of *Prkaa1*^WT^ and *Prkaa1*^ΔMφ^ male mice during 12-h light and dark cycles recorded on the second day after acclimatization. **(D)** Heat production of *Prkaa1*^WT^ and *Prkaa1*^ΔMφ^ male mice during 12-h light and dark cycles. **(E)** Daily food and drink intake of *Prkaa1*^WT^ and *Prkaa1*^ΔMφ^ male mice during light and dark cycles of animals fed HFD at room temperature (22°C). Area under the curve was calculated during light and dark cycles for each individual animal. RER is calculated by the ratio of VO_2_ and VCO_2_. Black horizontal bars denote the dark period of the day (12 h). AUC, area under the curve. All data are expressed as mean ± SEM. Statistical significance was determined by unpaired Student’s *t*-test. *p < 0.05 was considered significant, **p < 0.01, ***p < 0.001.

We next examined the metabolic rate and RER of these mice via assessments of oxygen consumption and carbon dioxide production using the Comprehensive Lab Animal Monitoring System (CLAMS). *Prkaa1*^ΔMφ^ mice showed higher oxygen consumption and carbon dioxide production than *Prkaa1*^WT^ mice, indicating a higher metabolic rate in the knockout mice (Figures 5A,B). However, there was no difference in RER (calculated by the ratio of CO_2_ production to O_2_ consumption), suggesting that myeloid *Prkaa1* deficiency may not affect energy substrate selection (Figure 5C). The heat production of *Prkaa1*^ΔMφ^ mice also trailed higher than those of *Prkaa1*^WT^ mice at both light and dark cycles (Figure 5D). In addition to higher energy expenditure, myeloid *Prkaa1* deficiency also exhibited lower energy intake as indicated by lower daily food and drink intake. As shown in Figure 5E, *Prkaa1*^ΔMφ^ mice showed decreased daily food intake compared to *Prkaa1*^WT^ mice. Although average daily drink has no significant difference between these two groups, the *Prkaa1*^ΔMφ^ mice still exhibited the trend of slightly lower drink intake than that of *Prkaa1*^WT^ mice. As such, these results indicate that myeloid *Prkaa1* increases energy expenditure and reduces energy intake in response to HFD-induced excessive nutrition.

### Myeloid *Prkaa1* Deficiency Decreases Western-Diet Induced Atherosclerosis

**FIGURE 6:**
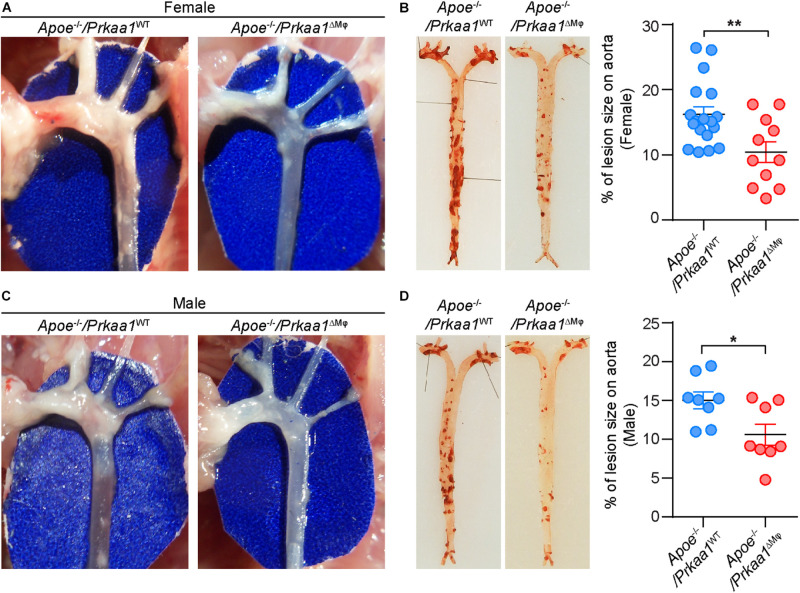
Myeloid *Prkaa1* deficiency decreases Western diet-induced atherosclerotic lesion size. **(A,C)** Aortic arches were dissected and photographed from *Apoe*^– /–^ /*Prkaa1*^WT^ and *Apoe*^– /–^ /*Prkaa1*^ΔMφ^ female and male mice fed with Western diet for 16 weeks. **(B,D)** Representative images and lesion area quantification of Oil Red O-stained-aortas (*en face*) from *Apoe*^– /–^ /*Prkaa1*^WT^ (female *n* = 18, male *n* = 8), *Apoe*^– /–^ /*Prkaa1*^ΔMφ^ (female *n* = 11, male *n* = 8) mice after being fed Western diet for 16 weeks. All data are expressed as mean ± SEM. Statistical significance was determined by unpaired Student’s *t*-test. *p < 0.05 was considered significant, **p < 0.01.

We also examined whether *Prkaa1* deficiency-mediated decreased leukocyte recruitment reduces diet-induced atherosclerosis. We bred *Prkaa1*^ΔMφ^ mice with *Apoe*^–/–^ mice and generated *Apoe*^–/–^/*Prkaa1*^ΔMφ^ atherosclerotic mice and their littermate *Apoe*^–/–^/*Prkaa1*^WT^ mice. Starting from 7 weeks of age, these age- and gender-matched mice were fed a Western diet containing 42% calories from fat and 0.2% (wt/wt) cholesterol for 16 weeks. *Apoe*^–/–^/*Prkaa1*^ΔMφ^ mice showed significantly fewer and smaller lesions at the aortic arches both in male and female mice than littermate control mice (Figures 6A,C). This observation was confirmed with quantitative measurement of atherosclerotic lesions following Oil Red O (ORO) staining of aortas (Figures 6B,D). Decreased levels of cholesterol, triglyceride, and glucose in blood of both male and female *Apoe*^–/–^/*Prkaa1*^ΔMφ^ mice were found (Supplementary Figure 5), likely contributing to the alleviated atherosclerosis in *Apoe*^–/–^/*Prkaa1*^ΔMφ^ mice. Additionally, *Apoe*^–/–^/*Prkaa1*^ΔMφ^ mice exhibited no significant difference in the number of circulating white blood cells, including neutrophils, leukocytes and monocytes, compared with *Apoe*^–/–^/*Prkaa1*^WT^ mice (Supplementary Figure 5).

**FIGURE 7:**
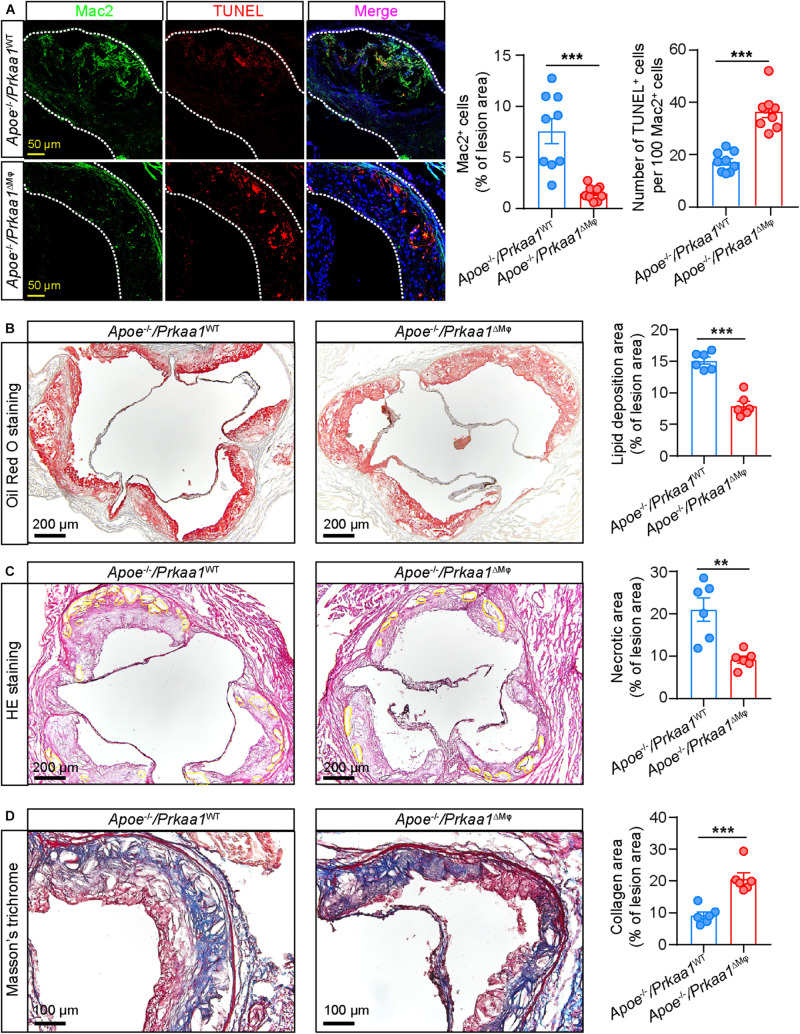
Myeloid *Prkaa1* deficiency improves features of plaque instability and decreases macrophage viability. **(A)** Representative images and quantification data of Mac2 and TUNEL staining of aortic sinuses from *Apoe*^– /–^ /*Prkaa1*^WT^, *Apoe*^– /–^ /*Prkaa1*^ΔMφ^ mice fed Western diet for 16 weeks. Apoptotic cells were labeled by TUNEL (Alexa Fluor-594, Red), macrophages were labeled by Mac2 staining (Alexa Fluor-488, green), and nuclei were counterstained with DAPI (blue). Scale bar: 50 μm; *n* = 9 mice per group. **(B)** Oil Red O staining of aortic sinuses of *Apoe*^– /–^ /*Prkaa1*^WT^ and *Apoe*^– /–^ /*Prkaa1*^ΔMφ^ male mice fed Western diet for 16 weeks, and quantification of lipid deposition. *n* = 6, Scale bar: 200 μm. **(C)** HE staining of necrotic core area in aortic sinuses of *Apoe*^– /–^ /*Prkaa1*^WT^ and *Apoe*^– /–^ /*Prkaa1*^ΔMφ^ male mice fed Western diet for 16 weeks, and percentages of necrotic core area. *n* = 6, Scale bar: 200 μm. **(D)** Masson trichrome staining of collagen in aortic sinuses of *Apoe*^– /–^ /*Prkaa1*^WT^ and *Apoe*^– /–^ /*Prkaa1*^ΔMφ^ male mice fed Western diet for 16 weeks, and percentages of collagen area. *n* = 6. Scale bar: 100 μm. All data are expressed as mean ± SEM. Statistical significance was determined by unpaired Student’s *t*-test. * *p* < 0.05 was considered significant, **p < 0.01, ***p < 0.001.

Aortic sinuses from these mice were further examined to evaluate the effect of *Prkaa1*-deficient myeloid cells in determining the size and stability of atherosclerotic lesions. Immunostaining of aortic sinus sections with antibody of the macrophage marker Mac2 showed that the number of macrophages was much less in *Apoe*^–/–^/*Prkaa1*^ΔMφ^ mice than control mice (Figure 7A). Additionally, macrophage viability in aortic sinuses of these mice was analyzed by TUNEL staining. The number of TUNEL-positive cells was markedly increased in the aortic sinuses of *Apoe*^–/–^/*Prkaa1*^ΔMφ^ mice compared with those of control mice (Figure 7A). These observations indicate that the decreased number of macrophages in lesions of *Apoe*^–/–^/*Prkaa1*^ΔMφ^ mice is due to both decreased recruitment of monocytes to arterial vessels and increased apoptosis of macrophages in atherosclerotic lesions. As a result, the size of atherosclerotic lesions (Figure 7B), indicated with ORO staining, was decreased while the stability of atherosclerotic lesions, evident with necrotic cores with HE staining and collagen content by Masson’s trichrome staining (Figures 7C,D), was increased in *Apoe*^–/–^/*Prkaa1*^ΔMφ^ mice compared to those of control mice. Thus, mice with myeloid deletion of *Prkaa1* exhibit less severe and more stable atherosclerosis.

## Discussion

In the present study, we found that *Prkaa1* expression is upregulated in leukocytes sorted from adipose tissue of HFD-fed mice, and this was accompanied with upregulated genes associated with glycolysis and FAO. Accordingly, compromised glycolysis and FAO was observed in *Prkaa1*-deficient macrophages. Leukocyte recruitment, evidenced with macrophage migration *in vitro* and leukocyte rolling and adhesion *in vivo*, was compromised in the absence of *Prkaa1.* Consequently, chronic inflammatory disorders, including diet-induced diabetes and atherosclerosis, were reduced in *Prkaa1*^ΔMφ^ mice.

AMPKα1/PRKAA1 mediates glycolysis and FAO in macrophages. AMPK/PRKA serves as a master regulator of energy metabolism to control key factors involved in the many pathways to maintain energy balance. In addition to hypoxia, other stresses such as chronic inflammation and aberrant glucose and lipid levels enhance and/or activate AMPK/PRKA. *Apoe* deficiency dysregulates the levels of glucose and lipids and causes chronic inflammation in mice. This may also result in increased function of AMPK. AMPK/PRKA maintains energy homeostasis by activating catabolic processes to increase ATP production and inhibiting anabolic processes to suppress ATP consumption. In order to replenish ATP stores, AMPK/PRKA actively stimulates glucose utilization by inducing glucose transporter expression and further increases glucose uptake into cells (Kim et al., 2010). AMPK/PRKA also has been reported to regulate glycolytic flux through the pathway by phosphorylating phosphofructo-2-kinase/fructose 2,6-bisphosphatase 3 (PFKFB3), which affects the activity of PFK1, a rate-limiting enzyme in glycolysis (Bando et al., 2005). Our previous study has demonstrated that AMPK/PRKA regulates glycolysis in part via the HIF1α pathway (Yang et al., 2018), and increased HIF1A expression promotes glycolysis, resulting in a rapid supply of ATP (Semenza, 2012). In addition to stimulating glycolysis, AMPK/PRKA also increases FAO by a reduction of the activity of ACC, decreased the level of malonyl-CoA, and resulted in increased fatty acid import into mitochondria for β-oxidation (Saggerson, 2008). The studies indicated that the AMPK–ACC–malonyl CoA–carnitine palmitoyl transferase 1 mechanism plays a key role in the physiological regulation of FAO (Dagher et al., 2001). Although these studies have indicated an interaction between AMPK/PRKA and cellular metabolism in cells of other types, our study has demonstrated that Prkaa1 is also critical in regulation of glycolysis and FAO in leukocytes in mice. Compromised production of ATP, especially on the actin compartment in *Prkaa1*-deficient leukocytes, may dramatically affect many steps of leukocyte recruitment, including rolling, adhesion and migration. Additionally, decreased energy production plus decreased metabolites of glycolysis and FAO may also result in compromised repair, leading to apoptosis of infiltrated macrophages.

The physiological effect of *Prkaa1* in leukocytes revealed in this study may not explain the pharmacological effect of AMPK/PRKA activation. Over the past decades, much work has emerged to support the beneficial role of AMPK in chronic inflammatory disorders such as metabolic syndrome and inflammatory diseases (Cheang et al., 2014; Guma et al., 2015; Kjøbsted et al., 2015). It has been reported that metformin or AICAR attenuates Ang II-induced atheromatous plaque formation and protects against hyperglycemia-induced atherosclerosis (Li et al., 2010; Vasamsetti et al., 2015; Wang et al., 2017) and also reduces insulin resistance (Yang et al., 2012). These beneficial effects may operate mainly through the effect on cells other than myeloid cells, such as vascular cells or metabolic cells. Also, some beneficial effect from the use of metformin, AICAR or A769662 may be through an AMPK-independent pathway (Guigas et al., 2006; Kirchner et al., 2018). Additionally, pharmacological activation of AMPKa1 in leukocytes may also reduce inflammation through many other pathways than the metabolism induced by pharmacologically activated AMPKa1. All of these possibilities will be explored in our future studies.

Phenotypic variance is noted in myeloid *Prkaa1-*deficient mice. Mice that were deficient in myeloid *Prkaa1*, when bred to *Ldlr*^–/–^ mice, displayed enhanced macrophage inflammation, increased plasma cholesterol and triglyceride levels, and a phenotype of enhanced diet-induced obesity and insulin resistance as well as accelerated atherosclerosis (Cao et al., 2016). In contrast, *Apoe*^–/–^ mice with *Prkaa1* deletion or myeloid *Prkaa1* deletion showed reduced monocyte differentiation and survival, thus attenuating the initiation and progression of atherosclerosis (Zhang et al., 2017). *Apoe*^–/–^ mice with myeloid *Prkaa2* deficiency developed smaller atherosclerotic plaques that contained fewer macrophages and less MMP9 than plaques from control mice through regulating myeloid DNA methylation (Fisslthaler et al., 2019). A very recent study did not find any difference in the size of atherosclerotic lesions between control *Apoe*^–/–^ mice and *Apoe*^–/–^ mice deficient in both *Prkaa1* and *Prkaa2* in myeloid cells (LeBlond et al., 2020). These inconsistent results from different studies indicate the disease phenotype is affected by many important experimental factors, such as background strain, littermate control, diet and the stages of disease when mice were evaluated. In our study, we have followed a recently published protocol for rodent atherosclerosis (Daugherty et al., 2017). With our expertise in cell metabolism and leukocyte recruitment, we are confident of our conclusion in which AMPK/PRKAA1/Prkaa1-mediated metabolism is critical for myeloid cell recruitment and survival.

## Conclusion

In conclusion, these findings clarify that AMPKα1 plays a key role in regulating macrophage glucose and lipid metabolism, further controls monocyte recruitment and macrophage viability, and eventually promotes the development of diet-induced insulin resistance and atherosclerosis. Targeting AMPKα1-driven macrophage metabolism could be a therapeutic intervention in cardiovascular diseases.

## Data Availability Statement

The raw data supporting the conclusions of this article will be made available by the authors, without undue reservation.

## Ethics Statement

The animal study was reviewed and approved by IACUC (Institutional Animal Care & Use Committee) of Augusta University.

## Author Contributions

QY, QM, and YH designed the experiments and co-wrote the manuscript. QY, QM, JX, JZ, SL, and ZL performed the experiments and prepared the figures and manuscript. QY, YZ, QD, ZL, and JS provided input on experimental design, methods, and research strategy. JZ, DF, and NW critically reviewed and revised the manuscript. ZB and MH provided the reagents or materials and participated in designing the experiments. All authors contributed to the article and approved the submitted version.

## Conflict of Interest

The authors declare that the research was conducted in the absence of any commercial or financial relationships that could be construed as a potential conflict of interest.

## Supplementary Material

Supplementary Figure 1 Schematic diagram of leukocyte sorting from adipose tissues. Stromal vascular cells (SVFs) were isolated from adipose tissues and stained with antibodies against CD45, CD31, and 7AAD. The cell subtypes identified using flow cytometry defined the leukocytes as CD45^+^ CD31^–^7AAD^–^.

Supplementary Figure 2 Representative images and quantification data of p-Prka and Pfkfb3 co-staining with F4/80 (macrophage markers) in adipose tissues from HFD- and CD-fed mice. *n* = 4, Scale bars, 20 μm. All data were expressed as mean ± SEM. Statistical significance was determined by unpaired Student’s *t*-test. ^∗^*p* < 0.05 was considered significant, ^∗∗^*p* < 0.01, ^∗∗∗^*p* < 0.001.

Supplementary Figure 3 **(A)** Schematic diagram of myeloid-specific Prkaa1-deficient mouse generation by crossing *Prkaa1*^*f/f*^ with *Lysm*^cre^ mice. **(B)** Representative genotyping gel demonstrating the generation of *Prkaa1* myeloid knockout mice. **(C)** Western blot analysis and densitometric quantification of Prkaa1 protein levels in BMDMs cultured from *Prkaa1*^WT^ and *Prkaa1*^ΔMφ^ mice. *n* = 6. All data are expressed as mean ± SEM. Statistical significance was determined by unpaired Student’s *t*-test. ^∗^*p* < 0.05 was considered significant, ^∗∗^*p* < 0.01, ^∗∗∗^*p* < 0.001.

Supplementary Figure 4 **(A)** Representative images and quantification of MCP-1-induced migration in BMDMs cultured from *Prkaa1*^WT^ and *Prkaa1*^ΔMφ^ mice in normal and high glucose conditions (NG: 5.5 mM, HG: 30 mM). *n* = 8, Scale bars, 100 μm. **(B)** Quantification data and representative images of flow cytometry analysis of Annexin V staining in BMDMs cultured from bone marrow of Prkaa1WT and Prkaa1ΔMφ mice in NG and HG conditions. *n* = 6. All data are expressed as mean ± SEM. Statistical significance was determined by one-way ANOVA followed by Bonferroni test. ^∗^*p* < 0.05 was considered significant, ^∗∗^*p* < 0.01, ^∗∗∗^*p* < 0.001.

Supplementary Figure 5 **(A)** Levels of total cholesterol in plasma of *Apoe*^–/–^/*Prkaa1*^WT^ (female, *n* = 8; male, *n* = 8), *Apoe*^–/–^/*Prkaa1*^ΔMφ^ (female, *n* = 7; male, *n* = 7) mice fed Western diet for 16 weeks. **(B)** Levels of triglyceride in plasma of *Apoe*^–/–^/*Prkaa1*^WT^ (female, *n* = 7; male, *n* = 8), *Apoe*^–/–^/*Prkaa1*^ΔMφ^ (female, *n* = 5; male, *n* = 7) mice fed Western diet for 16 weeks. **(C)** Levels of glucose in plasma of *Apoe*^–/–^/*Prkaa1*^WT^ (*Prkaa1*^ΔMφ^, female, *n* = 7; male, *n* = 9) mice fed Western diet for 16 weeks. All data are expressed as mean ± SEM. Statistical significance was determined by unpaired Student’s *t*-test. ^∗^*p* < 0.05 was considered significant, ^∗∗^*p* < 0.01, ^∗∗∗^*p* < 0.001.

Supplementary Figure 6 Hemavet data analysis of white blood cells (WBC), neutrophils (NE), leukocytes (LY) and monocytes (MO) from *Apoe*^–/–^/*Prkaa1*^WT^ (female, *n* = 13; male, *n* = 15), *Apoe*^–/–^/*Prkaa1*^ΔMφ^ (female, *n* = 14; male, *n* = 11) mice fed Western diet for 16 weeks. All data are expressed as mean ± SEM. Statistical significance was determined by unpaired Student’s *t*-test. ^∗^*p* < 0.05 was considered significant, ^∗∗^*p* < 0.01, ^∗∗∗^*p* < 0.001. (PLT, primed lymphocyte typing).
