# Supplementary figures and images for: Prkaa1 Metabolically Regulates Monocyte/Macrophage Recruitment and Viability in Diet-Induced Murine Metabolic Disorders
Source: Front Cell Dev Biol. 2021 Jan 12;8:611354. doi: 10.3389/fcell.2020.611354 (PMC7835533; doi:10.3389/fcell.2020.611354)

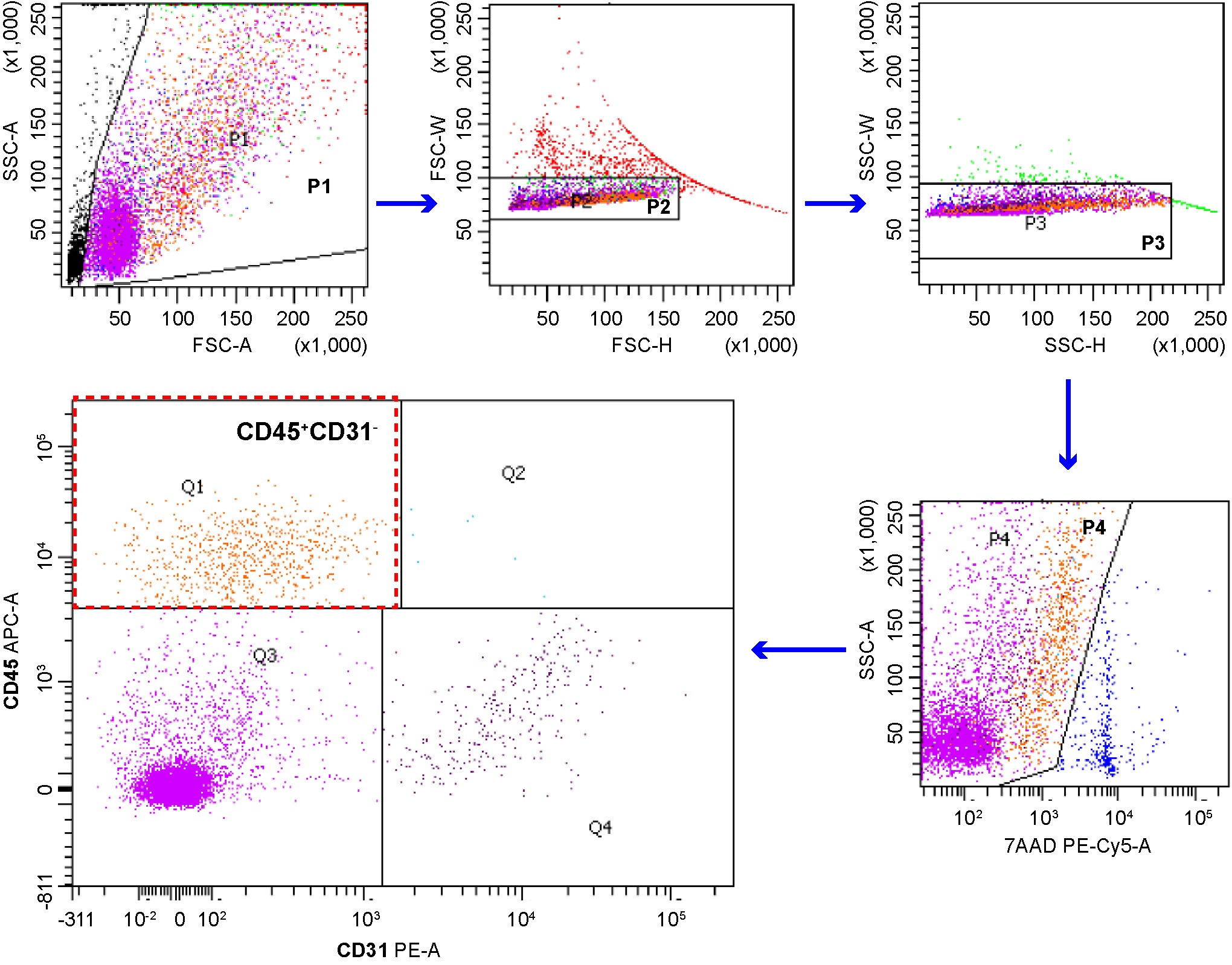

Supplement: Supplementary Figure 1 — Schematic diagram of leukocyte sorting from adipose tissues. Stromal vascular cells (SVFs) were isolated from adipose tissues and stained with antibodies against CD45, CD31, and 7AAD. The cell subtypes identified using flow cytometry defined the leukocytes as CD45+ CD31–7AAD–. [file Image_1.TIF]

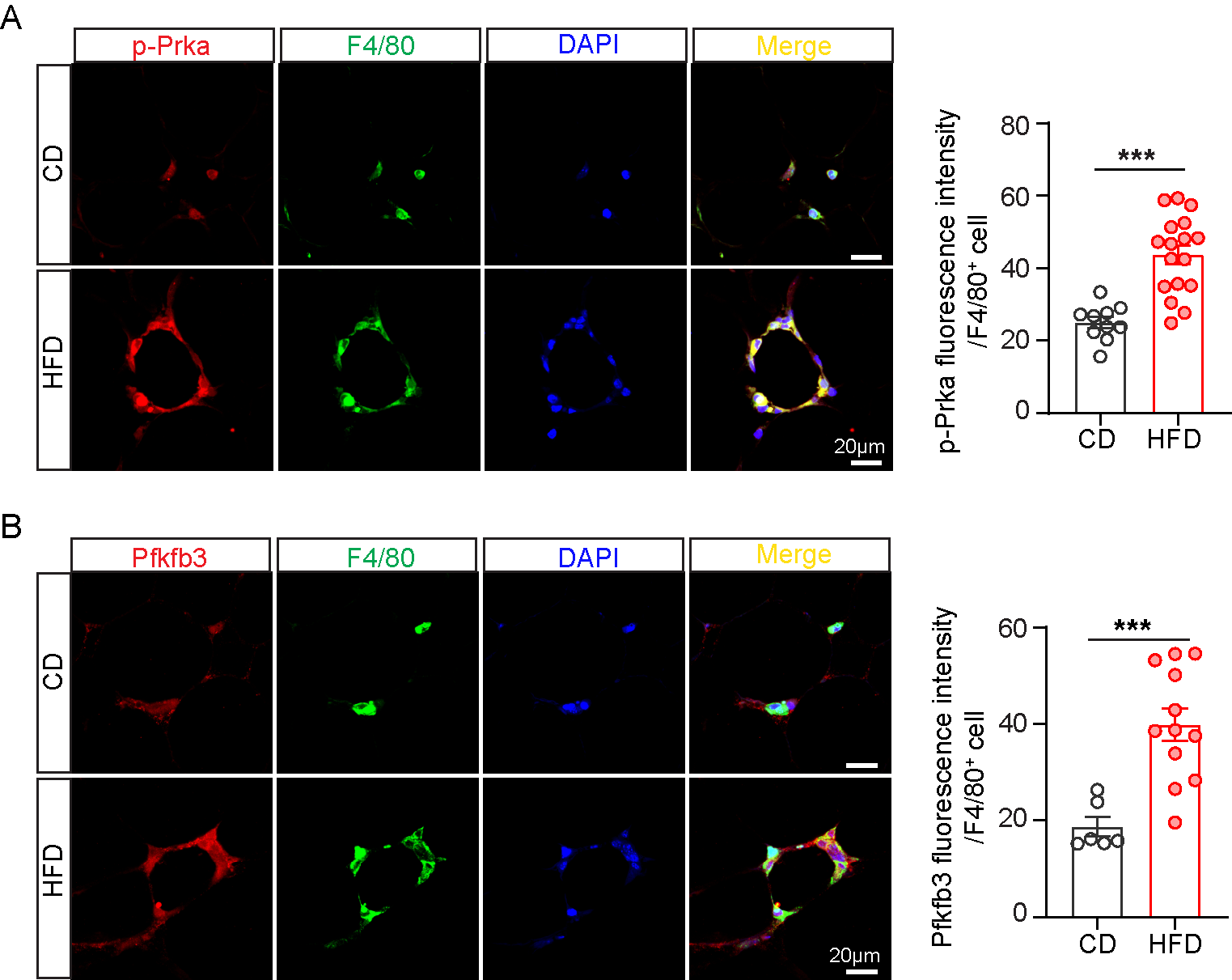

Supplement: Supplementary Figure 2 — Representative images and quantification data of p-Prka and Pfkfb3 co-staining with F4/80 (macrophage markers) in adipose tissues from HFD- and CD-fed mice. n = 4, Scale bars, 20 μm. All data were expressed as mean ± SEM. Statistical significance was determined by unpaired Student’s t-test. ∗p < 0.05 was considered significant, ∗∗p < 0.01, ∗∗∗p < 0.001. [file Image_2.TIF]

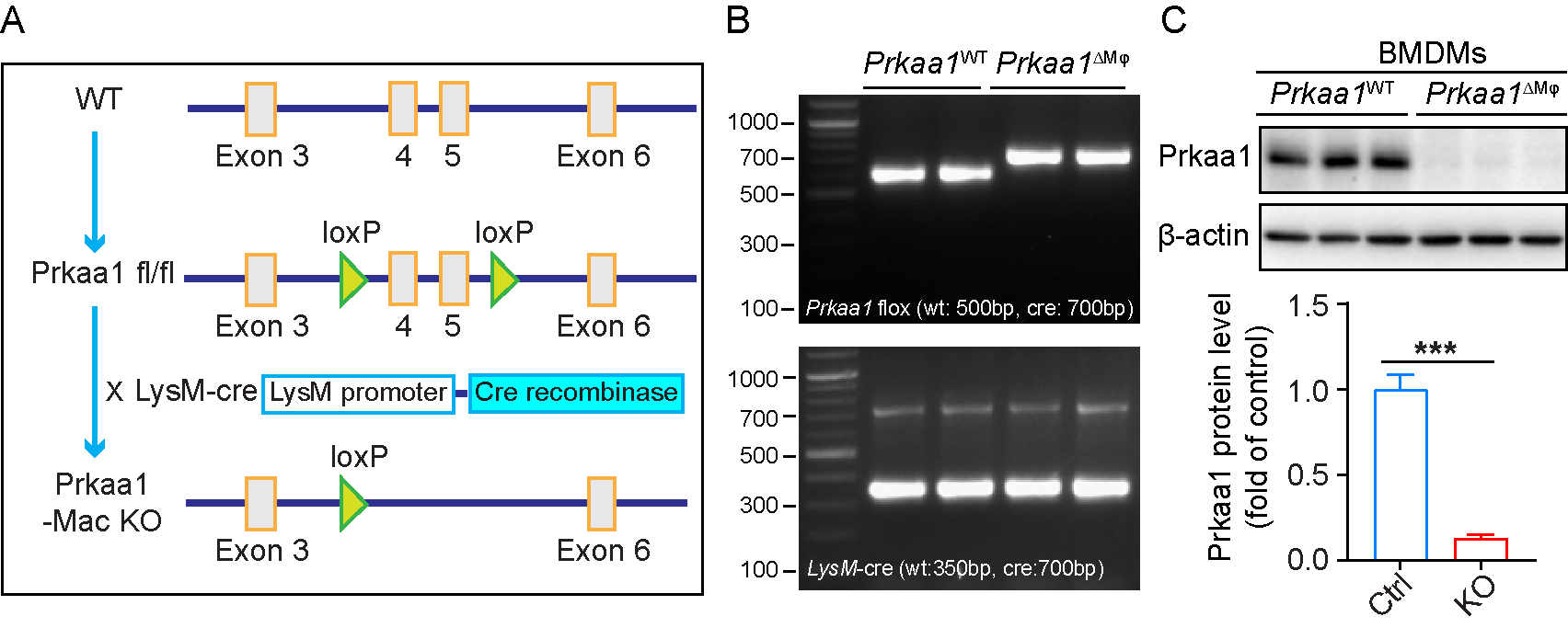

Supplement: Supplementary Figure 3 — (A) Schematic diagram of myeloid-specific Prkaa1-deficient mouse generation by crossing Prkaa1f/f with Lysmcre mice. (B) Representative genotyping gel demonstrating the generation of Prkaa1 myeloid knockout mice. (C) Western blot analysis and densitometric quantification of Prkaa1 protein levels in BMDMs cultured from Prkaa1WT and Prkaa1ΔMφ mice. n = 6. All data are expressed as mean ± SEM. Statistical significance was determined by unpaired Student’s t-test. ∗p < 0.05 was considered significant, ∗∗p < 0.01, ∗∗∗p < 0.001. [file Image_3.TIF]

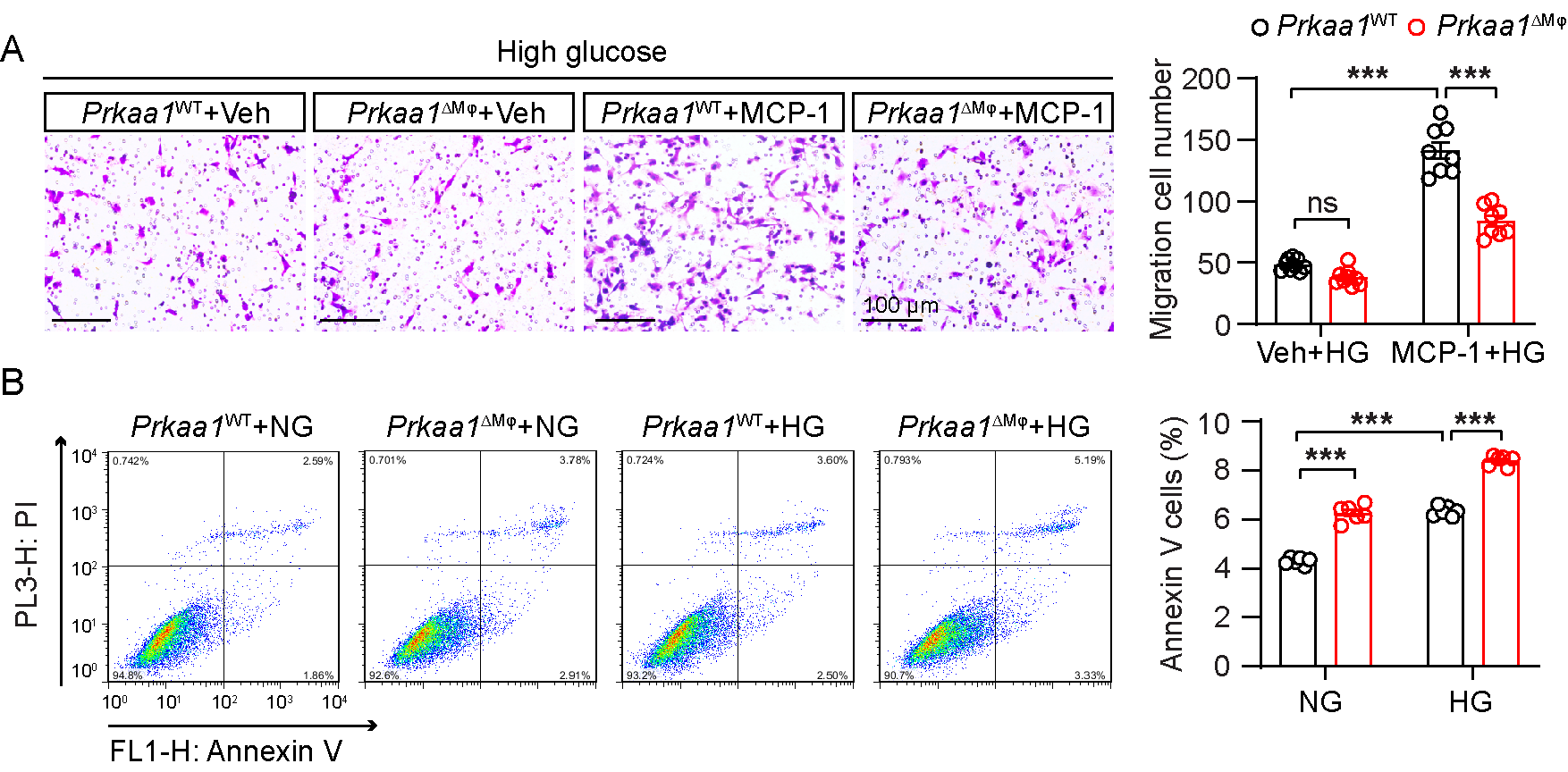

Supplement: Supplementary Figure 4 — (A) Representative images and quantification of MCP-1-induced migration in BMDMs cultured from Prkaa1WT and Prkaa1ΔMφ mice in normal and high glucose conditions (NG: 5.5 mM, HG: 30 mM). n = 8, Scale bars, 100 μm. (B) Quantification data and representative images of flow cytometry analysis of Annexin V staining in BMDMs cultured from bone marrow of Prkaa1WT and Prkaa1ΔMφ mice in NG and HG conditions. n = 6. All data are expressed as mean ± SEM. Statistical significance was determined by one-way ANOVA followed by Bonferroni test. ∗p < 0.05 was considered significant, ∗∗p < 0.01, ∗∗∗p < 0.001. [file Image_4.TIF]

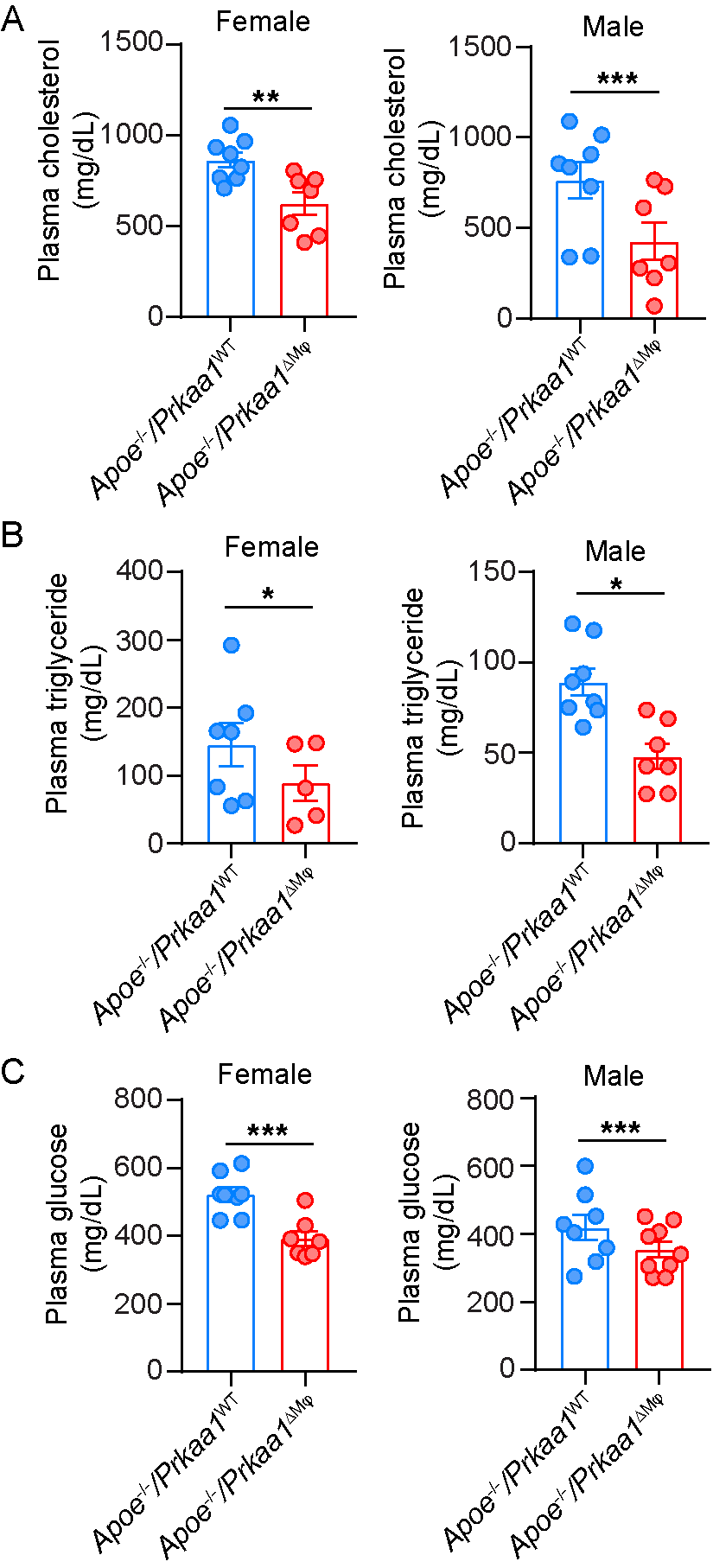

Supplement: Supplementary Figure 5 — (A) Levels of total cholesterol in plasma of Apoe–/–/Prkaa1WT (female, n = 8; male, n = 8), Apoe–/–/Prkaa1ΔMφ (female, n = 7; male, n = 7) mice fed Western diet for 16 weeks. (B) Levels of triglyceride in plasma of Apoe–/–/Prkaa1WT (female, n = 7; male, n = 8), Apoe–/–/Prkaa1ΔMφ (female, n = 5; male, n = 7) mice fed Western diet for 16 weeks. (C) Levels of glucose in plasma of Apoe–/–/Prkaa1WT (Prkaa1ΔMφ, female, n = 7; male, n = 9) mice fed Western diet for 16 weeks. All data are expressed as mean ± SEM. Statistical significance was determined by unpaired Student’s t-test. ∗p < 0.05 was considered significant, ∗∗p < 0.01, ∗∗∗p < 0.001. [file Image_5.TIF]

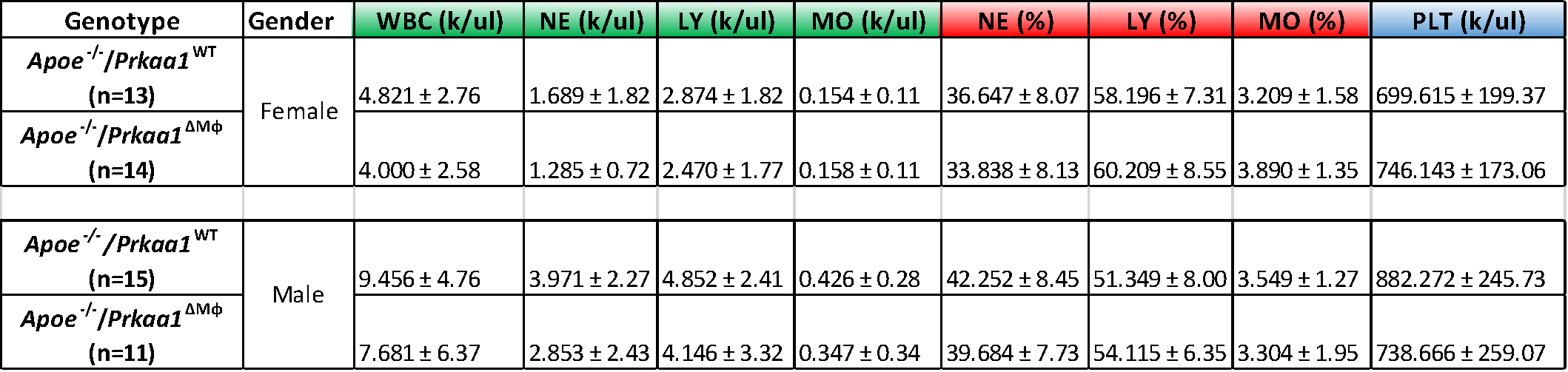

Supplement: Supplementary Figure 6 — Hemavet data analysis of white blood cells (WBC), neutrophils (NE), leukocytes (LY) and monocytes (MO) from Apoe–/–/Prkaa1WT (female, n = 13; male, n = 15), Apoe–/–/Prkaa1ΔMφ (female, n = 14; male, n = 11) mice fed Western diet for 16 weeks. All data are expressed as mean ± SEM. Statistical significance was determined by unpaired Student’s t-test. ∗p < 0.05 was considered significant, ∗∗p < 0.01, ∗∗∗p < 0.001. (PLT, primed lymphocyte typing). [file Image_6.TIF]
